# Impact of standardized immunophenotyping and MRD monitoring on early mortality reduction in childhood leukemia: a step towards addressing healthcare disparities in vulnerable populations from Southern Mexico

**DOI:** 10.3389/fonc.2025.1614445

**Published:** 2025-07-28

**Authors:** Laura Alfaro-Hernández, Dalia Ramírez-Ramírez, Rubí Romo-Rodríguez, Karen Ayala-Contreras, Ángeles del Campo-Martínez, Enrique López-Aguilar, Janet Flores Lujano, Aldo Allende-López, Erika Alarcón-Ruiz, Ma Del Rocio Banos-Lara, Diana Casique-Aguirre, Jesús Elizarrarás-Rivas, Javier Antonio López-Aquino, Miguel Ángel Garrido-Hernández, Daniela Olvera-Caraza, Vanessa Terán-Cerqueda, Juan Carlos Solís-Poblano, Pierre Mitchel Aristil-Chery, Enoch Alvarez-Rodríguez, Wilfrido Herrera-Olivares, Lénica Anahí Chavez-Aguilar, Aquilino Márquez-Toledo, Lena Sarahi Cano-Cuapio, Nuria Citlalli Luna-Silva, Maria Angélica Martínez-Martell, Anabel Beatriz Ramirez-Ramirez, Juan Carlos Rodríguez-Espinosa, Daniela Medina-León, Roberto Rodríguez-Díaz, Minerva Mata-Rocha, Amanda Idaric Olivares-Sosa, Haydeé Rosas-Vargas, Juan Manuel Mejia-Arangure, Lourdes Millán-Pérez-Peña, Rosana Pelayo, Juan Carlos Núñez-Enríquez

**Affiliations:** ^1^ Laboratorio de Citómica del Cáncer Infantil, Centro de Investigación Biomédica de Oriente, Instituto Mexicano del Seguro Social, Puebla, Mexico; ^2^ Facultad de Ciencias Químicas, Benemérita Universidad Autónoma de Puebla, Puebla, Mexico; ^3^ Coordinación de Atención Oncológica, Instituto Mexicano del Seguro Social, Mexico City, Mexico; ^4^ Unidad de Investigación Médica en Epidemiología Clínica, Unidad Médica de Alta Especialidad (UMAE) Hospital de Pediatría “Dr. Silvestre Frenk Freund”, Centro Médico Nacional Siglo XXI, Instituto Mexicano del Seguro Social (IMSS), Mexico City, Mexico; ^5^ Tecnológico Nacional de México, Instituto Tecnológico de Ciudad de Madero, Ciudad Madero, Tamaulipas, Mexico; ^6^ Facultad de Medicina, Universidad Popular Autónoma del Estado de Puebla, Puebla, Mexico; ^7^ Centro de Investigación Oncológica Una Nueva Esperanza, Universidad Popular Autónoma del Estado de Puebla, Puebla, Mexico; ^8^ Secretaría de Ciencia, Humanidades, Tecnología e Innovación (Secihti), Mexico City, Mexico; ^9^ Coordinación de Investigación en Salud, Instituto Mexicano del Seguro Social, Oaxaca, Mexico; ^10^ Coordinación Clínica de Educación e Investigación en Salud de la Unidad de Medicina Familiar (UMF) No. 1, Instituto Mexicano del Seguro Social, Oaxaca, Mexico; ^11^ Servicio de Oncohematología Pediátrica, Hospital para el Niño Poblano, Secretaria de Salud (SS), Puebla, Mexico; ^12^ Servicio de Oncohematología Pediátrica, Instituto Mexicano del Seguro (IMSS) Unidad Médica de Alta Especialidad (UMAE) Centro Médico Nacional (CMN) Hospital de Especialidades Dr. Manuel Ávila Camacho, Puebla, Mexico; ^13^ Servicio de Hematología, Instituto Mexicano del Seguro (IMSS) Unidad Médica de Alta Especialidad (UMAE) Centro Médico Nacional (CMN) Hospital de Especialidades Dr. Manuel Ávila Camacho, Puebla, Mexico; ^14^ Departamento de Enseñanza e Investigación, Instituto de Seguridad y Servicios Sociales de los Trabajadores al Servicio de los Poderes del Estado de Puebla (ISSSTEP), Puebla, Mexico; ^15^ Servicio de Oncohematología Pediátrica, Instituto de Seguridad y Servicios Sociales de los Trabajadores al Servicio de los Poderes del Estado de Puebla (ISSSTEP), Puebla, Mexico; ^16^ Servicio de Hematología Pediátrica, Instituto de Seguridad y Servicios Sociales de los Trabajadores del Estado (ISSSTE), Puebla, Mexico; ^17^ Servicio de Oncohematología, Hospital General del Sur Dr. Eduardo Vázquez Navarro, Secretaria de Salud (SS), Puebla, Mexico; ^18^ Departamento de Pediatría, Hospital Universitario de Puebla (BUAP), Puebla, Mexico; ^19^ Servicio de Oncología Pediátrica, Hospital Infantil de Tlaxcala, Secretaria de Salud (SS), Tlaxcala, Mexico; ^20^ Servicio de Hemato-Oncología Pediátrica, Hospital de la Niñez Oaxaqueña “Dr. Guillermo Zárate Mijangos”, Secretaria de Salud y Servicios de Salud Oaxaca (SSO), Oaxaca, Mexico; ^21^ Servicio de Oncocrean, Hospital General de Zona 01 “Dr. Demetrio Mayoral Pardo” Instituto Mexicano del Seguro Social (IMSS), Oaxaca, Mexico; ^22^ Facultad Mexicana De Medicina Universidad La Salle, Mexico City, Mexico; ^23^ Facultad de Medicina, Universidad Nacional Autónoma de México (UNAM), México City, Mexico; ^24^ Unidad de Investigación Médica en Genética Humana, Unidad Médica de Alta Especialidad (UMAE) Hospital de Pediatría “Dr. Silvestre Frenk Freund”, Centro Médico Nacional Siglo XXI, Instituto Mexicano del Seguro Social (IMSS), Mexico City, Mexico; ^25^ Dirección de Educación e Investigación, Unidad Médica de Alta Especialidad (UMAE) Hospital de Pediatría “Dr. Silvestre Frenk Freund”, Centro Médico Nacional Siglo XXI, Instituto Mexicano del Seguro Social (IMSS), Mexico City, Mexico; ^26^ Laboratorio de Genómica Funcional del Cáncer, Instituto Nacional de Medicina Genómica (INMEGEN), Mexico City, Mexico; ^27^ Laboratorio de Bioquímica y Biología Molecular, Centro de Química, Instituto de Ciencias, Benemérita Universidad Autónoma de Puebla, Puebla, Mexico; ^28^ Unidad de Educación e Investigación, Instituto Mexicano del Seguro Social, Mexico City, Mexico; ^29^ División de Investigación en Salud, Unidad Médica de Alta Especialidad (UMAE) Hospital de Pediatría “Dr. Silvestre Frenk Freund”, Centro Médico Nacional Siglo XXI, Instituto Mexicano del Seguro Social (IMSS), Mexico City, Mexico

**Keywords:** pediatric B-cell acute lymphoblastic leukemia, immunophenotyping, measurable residual disease (MRD), early-mortality, low-and middle -income countries

## Abstract

**Background:**

Despite high cure rates for pediatric B-cell acute lymphoblastic leukemia (B-ALL) in high-income countries, early mortality remains unacceptably high in low- and middle-income countries (LMICs), largely due to limited access to risk-adapted therapy and response monitoring. Southern Mexico, a region marked by socioeconomic vulnerability, is emblematic of this disparity. In 2022 the Childhood Cancer Cytomics Laboratory (CCCL) started the implementation of standardized protocols for immunophenotyping and measurable residual disease (MRD) monitoring of B-ALL patients from this region.

**Objective:**

To evaluate the impact of implementing standardized immunophenotyping and MRD monitoring on early mortality in children with B-ALL treated in public hospitals in southern Mexico.

**Methods:**

A prospective cohort study was conducted between 2022 and 2024. Before initiating CCCL activities, public hospitals were invited to participate, and standardized protocols for sample collection, handling, and transportation were implemented across all participating sites. A total of 298 children newly diagnosed with B-ALL were enrolled and followed throughout treatment. Patients were stratified based on whether immunophenotyping and MRD monitoring were performed at the CCCL. MRD was assessed at the end of induction (EOI) therapy using standardized EuroFlow-based flow cytometry protocols. Simultaneously, early mortality—defined as death occurring within the first year after diagnosis—was evaluated. Adjusted hazard ratios (aHR) and 95% confidence intervals (CI) were estimated using multivariable Cox regression, with p-values < 0.05 considered statistically significant.

**Results:**

Early mortality was significantly lower among patients who underwent MRD monitoring at the CCCL (10.8%) compared to those who did not (24.8%, p<0.01). One-year overall survival was also higher in patients evaluated at the CCCL (89.6% *vs*. 75.2%, p<0.001). In the multivariable Cox regression analysis, patients who underwent MRD monitoring at the CCCL showed a significantly lower risk of early mortality during the first year of treatment (adjusted hazard ratio [aHR] 0.41; 95% CI: 0.22–0.77; p < 0.01), after adjusting for sex, NCI risk classification, treatment abandonment, and early relapse. MRD positivity was associated with a CD34^+^ ProB immunophenotype, suggesting a more treatment-resistant leukemic profile.

**Conclusion:**

Centralized, standardized MRD monitoring at the CCCL was associated with a reduction in early mortality and improved one-year survival in children with B-ALL from a socioeconomically vulnerable population. These findings demonstrate the clinical value and feasibility of implementing MRD-informed response assessment in LMICs and highlight the potential of centralized diagnostic platforms to reduce survival disparities in childhood leukemia.

## Introduction

Over the past decades, the integration of biologically informed strategies and risk-adapted therapeutic protocols has transformed the prognosis of B-cell acute lymphoblastic leukemia (B-ALL), particularly in pediatric populations (1).

In high-income countries, five-year event-free survival rates now reach 85–90% among children diagnosed with B-ALL. However, outcomes in low- and middle-income countries (LMICs) remain considerably lower, primarily due to persistent barriers to effective care (2). These include severe socioeconomic challenges, limited geographic access to specialized healthcare services, and inadequate availability of diagnostic technologies and tools for monitoring treatment response (3).

Mexico is among the Latin American countries with the highest incidence and mortality rates of childhood leukemia (4–7). Previous studies have reported alarmingly high early mortality rates, particularly within the first year following diagnosis. These outcomes are largely attributed to chemotherapy-related toxicity and early relapses, often resulting from imprecise diagnosis, inadequate risk stratification, and the absence of standardized monitoring of treatment response—especially within public healthcare institutions (8–11).

It is well established that accurate and timely diagnosis, combined with systematic monitoring of treatment response, are critical components for effective risk stratification and therapy adjustment in pediatric B-ALL (12). These interventions are essential to prevent complications related to chemotherapy toxicity, minimize the risk of relapse due to under-treatment, and ultimately reduce mortality (13).

Measurable residual disease (MRD) quantification by flow cytometry immunophenotyping or polymerase chain reaction assessment of aberrant TCR or IGH rearrangements represents the most powerful and independent prognostic marker for both short- and long-term clinical outcomes in childhood B-ALL (14–16).

The objective of this study was to evaluate the impact of implementing standardized immunophenotyping and MRD monitoring on early mortality in pediatric patients with B-ALL in southern Mexico—a region characterized by pronounced socioeconomic vulnerability, including high poverty rates and limited access to healthcare services, particularly when compared to the northern regions of the country (9).

## Methods

We conducted a prospective cohort study from March 1, 2022, to December 31, 2024, coinciding with the implementation and consolidation of standardized protocols for immunophenotyping and MRD monitoring at the Childhood Cancer Cytomics Laboratory (CCCL). These efforts were part of a broader initiative called *National Project for Research and Incidence of Childhood Leukemias (PRONAII)*, aimed at establishing a comprehensive roadmap for the diagnosis, follow-up, and research of childhood leukemias in vulnerable regions of Mexico. This initiative aims to create a significant impact on clinical practice and public policy by enhancing prevention, diagnosis, and treatment of the disease. The methodology was recently published (9).

The institution of the CCCL at the Centro de Investigación Biomédica de Oriente (CIBIOR) of the Mexican Social Security Institute (IMSS) adhered to ethical guidelines for the handling of biological samples and sensitive data. Bone marrow (BM) samples were collected in compliance with protocols accepted by local ethics and research committees, ensuring full adherence to the Declaration of Helsinki. The study was approved by the ethics committees of all participating centers, and written informed consent to participate in this study was provided by the participants legal guardian/next of kin.

The CCCL implemented sample management protocols. Later on, these standards were formally communicated to all participating public hospitals prior to the initiation of CCCL activities (9). Notably, no patient was excluded from treatment of from inclusion in the study based on the completeness of immunophenotyping and MRD sample processing at CCCL.

### Patient recruitment, diagnosis confirmation and cohort follow-up

We analyzed data from newly diagnosed B-ALL patients under 19 years of age, who were identified in the participating hospitals between March 1, 2022, and December 31, 2023. Patients with Down syndrome or those who were not treated at the participating hospitals were excluded from the analysis. Patient recruitment was carried out by trained fieldworkers assigned to each participating hospital, who identified new leukemia cases. Parents were approached and invited to participate in the study. The diagnosis of B-ALL was validated by pediatric hematologists/oncologists based on clinical presentation and confirmed through bone marrow aspirate analysis, which included morphological assessment, immunophenotyping, and, when available, cytogenetic and molecular studies, in accordance with the 2008 World Health Organization (WHO) classification of lymphoid neoplasms. Cohort follow-up began on the date of B-ALL diagnosis confirmation (Day 0) and continued until the date of the last hospital visit or death. All patients were monitored for a minimum of one year following diagnosis confirmation.

Participating hospitals included the Hospital de la Niñez Oaxaqueña and Hospital General de Zona 01 “Dr. Demetrio Mayoral Pardo” (Oaxaca, Mexico), Hospital Infantil de Tlaxcala (Tlaxcala, Mexico), Hospital para el Niño Poblano, and the Unidad Médica de Alta Especialidad “Manuel Ávila Camacho” of the IMSS, Instituto de Seguridad y Servicios Sociales de los Trabajadores al Servicio de los Poderes del Estado de Puebla (ISSSTEP), Instituto de Seguridad y Servicios Sociales de los Trabajadores del Estado (ISSSTE), and Instituto de Seguridad y Servicios Sociales de los Trabajadores del Estado (ISSSTE), (Puebla, Mexico).

### Chemotherapy protocol

All participating hospitals adhered to a chemotherapy protocol for B-ALL based on the St. Jude Total XV regimen, which is structured into three sequential phases: Remission Induction (42 days), Consolidation (8 weeks), and Maintenance (120 weeks), the latter incorporating two risk-adapted reinduction blocks. The Remission Induction phase is based on the four cornerstone drugs historically used in ALL treatment: prednisone (40 mg/m²/day orally), vincristine (1.5 mg/m² weekly for four doses), daunorubicin (25 mg/m² weekly for two doses), and L-asparaginase (10,000 IU/m² every 48 hours for 6 to 9 doses). This is followed by Induction Phase B, involving three drugs: cyclophosphamide (1 g/m² single dose), cytarabine (75 mg/m²/day for 8 doses), and 6-mercaptopurine (60 mg/m²/day for 14 days). The Consolidation phase consists of high-dose methotrexate tailored to the patient’s assigned risk category: 2.5 g/m² for low-risk and 5 g/m² for standard- and high-risk patients, administered every 14 days for four doses. This is accompanied by CNS-directed intensification with four doses of intrathecal chemotherapy and daily 6-mercaptopurine (50 mg/m²/day). The Maintenance phase spans 120 weeks and includes multidrug therapy, featuring two risk-adapted reinduction blocks during weeks 7–9 and 17–19, respectively.

### Study variables and definitions

All clinical data were obtained from the patients’ medical records. The variables analyzed included: patient’s sex, age at diagnosis, National Cancer Institute (NCI) risk classification (standard risk, high risk), central nervous system (CNS) and/or testicular infiltration at diagnosis, gene rearrangements (when available), years of maternal education, public health insurance affiliation (yes/no), death during induction (yes/no), early mortality (yes/no), and causes of death.

### NCI risk classification

The risk was assigned based on age and peripheral white blood cell (WBC) count at diagnosis. Patients aged between 1.00 and 9.99 years with a WBC count < 50 × 10^9^/L were classified as standard risk. In contrast, those aged ≤ 1 year or ≥ 10 years and/or with a WBC count ≥ 50 × 10^9^/L were classified as high risk, in accordance with established criteria (17).

Complete remission was defined as the presence of < 5% bone marrow blasts by microscopy without evidence of extramedullary disease and to be determined at end of induction.

Induction failure (IF) was considered if overt leukemia was present, defined as more than 5% blasts in the marrow at the end of the induction phase, as determined by morphologic examination. Patients who achieved morphologic remission with <5% blasts and clearance of extramedullary disease but developed disease recurrence during the first year of treatment were classified as in relapse. Bone marrow relapse was defined by ≥25% morphologic blasts or ≥5% blasts with concomitant extramedullary relapse. Central nervous system (CNS) relapse was identified by CNS3 status (≥5 WBC/microliter cerebrospinal fluid with blasts on cytospin) or clinical signs of CNS leukemia. Isolated extramedullary recurrence had to be confirmed by biopsy.

Early mortality was considered as death occurring within the first year after diagnosis confirmation. One-year overall survival (OS) was defined based on death status (yes/no) within the first 12 months following diagnosis confirmation.

Maternal years of education were used as an indicator of socioeconomic status, following the Childhood Leukemia International Consortium (CLIC) classification (0–9 years, 9.1–12.9 years, ≥13 years of education) (18).

### BM sample collection, processing and flow cytometry analysis performed at the CCCL

BM aspirate samples from patients were analyzed at two different key points: at the time of B-ALL diagnosis and at the end of the induction (EOI) (day 42), during follow-up.

BM cell staining was performed as previously described, following the EuroFlow™ standard operating procedures (SOP) for flow cytometry analysis (19). The fluorochrome panel used for immunophenotyping and MRD assessment is detailed in **Supplementary Table 1**. Initially, samples were stained using the Acute Leukemia Orientation Tube (ALOT) to identify the lineage of immature blast cell populations. Additionally, an extended antibody panel was employed to characterize the malignant cell population.

For MRD monitoring, BM samples from diagnosed patients were processed according to the EuroFlow™ bulk-lysis SOP. Samples were stained with the EuroFlow™ 8-color BCP-ALL MRD antibody panel (20). Acquisition was carried out using BD FACSCanto II or BD FACSLyric cytometers. For the quantification of MRD, all cellular events obtained through MRD-specific immunophenotypic staining were acquired and analyzed. MRD detection was performed using a reference population of 5 × 10^6^ nucleated bone marrow cells, in accordance with the standardized guidelines established by the EuroFlow Consortium.

Data analysis was performed using Infinicyt software (version 2.0.6 CE-IVD, Cytognos SL, Salamanca, Spain), and the relative distribution of stained cells in the BM was determined to calculate the percentage of positive cells among all BM nucleated cells. The gating strategy used to identify leukemic cell populations and exclude normal counterparts is detailed in the **Supplementary Material** (**Supplementary Figures S1, S2**). B-ALL was classified into three categories based on CD34 expression and cell lineage: ProB-ALL (CD34^+^ CD19^+^), ProB-PreB-ALL (CD34^-/+^ CD19^+^), and PreB-ALL (CD34^-^ CD19^+^).

### Statistical analysis

For the present analysis patients were classified into two groups according to the completeness of immunophenotyping and measurable residual disease (MRD) evaluation performed at the CCCL, based on the processing and analysis of both diagnostic and end-of-induction bone marrow samples.

MRD CCCL-Yes: This group comprised patients whose diagnostic and end-of-induction samples were fully processed and analyzed at CCCL in accordance with standardized protocols established by the CCCL.

MRD CCCL-No: This group included patients for whom samples were either unavailable or not processed and/or analyzed at CCCL, thereby precluding complete MRD assessment within the centralized framework.

Descriptive statistics were used to summarize the demographic and clinical characteristics of the study population. Categorical variables were expressed as absolute frequencies and corresponding percentages. Group comparisons for categorical variables were performed using the Chi-square test or Fisher’s exact test when appropriate.

The primary outcome was early mortality, defined as death occurring within the first year of treatment. This outcome was compared between groups using bivariate analyses to identify potential associations. A p-value of <0.05 was considered statistically significant. All statistical tests were two-sided.

Additionally, overall survival (OS) during the first year from diagnosis was assessed between groups based on whether MRD testing was performed at the CCCL or not. Kaplan-Meier survival curves were generated to estimate the survival probabilities in both groups, and comparisons between the groups were made using the log-rank test. A significance level of p < 0.05 was used for the log-rank test to determine if there were statistically significant differences in survival between the MRD and non-MRD groups, providing further insight into the impact of MRD testing on early mortality.

A multivariable Cox proportional hazards regression model was performed to evaluate the association between undergoing MRD monitoring at the CCCL and early mortality, adjusting for potential confounders such as patient sex, NCI risk classification, treatment abandonment, and early relapse. Adjusted hazard ratios (aHR) with 95% confidence intervals (CI) for each variable were calculated.

Data were analyzed using SPSS version 27, and results are presented in accordance with STROBE guidelines for observational studies (21).

## Results

A total of 298 pediatric patients with B-ALL were included in the study, representing 100% of newly diagnosed cases at the participating hospitals. Of these, 129 patients (43.3%) underwent MRD testing at the CCCL, while 169 (56.7%) did not.

Within the study cohort, a subset of patients lacked complete MRD evaluation due to the absence of diagnostic and/or end-of-induction bone marrow samples processed at the CCCL. The underlying causes of incomplete MRD monitoring reflected real-world operational and clinical barriers. These included insufficient sample volume or inadequate sample quality, technical malfunctions in local flow cytometry platforms, inability to transport or process samples during weekends or holidays, diagnostic assessments performed at external institutions, lack of informed parental consent, institutional or clinician preference for alternative laboratories, and patient clinical instability at the time of sampling.

As shown in **Table 1**, there were no statistically significant differences between groups in terms of sex distribution, age at diagnosis, or maternal education level.

**Table 1:** Baseline characteristics of the study population stratified by MRD testing at CCCL.

Study variables	MRD testing at CCCL		P-value*
	Yes (n=129)	No (n=169)	
	n (%)	n (%)	
Patient´s sex			
Male	74 (57.4)	87 (51.5)	0.31
Female	55 (42.6)	82 (48.5)	
Patient´s Age at Diagnosis			
< 1 year	1 (0.7)	2 (1.2)	0.88
1–4 years	42 (32.5)	48 (28.4)	
5–9 years	34 (26.4)	43 (25.4)	
10–14 years	34 (26.4)	53 (31.4)	
15–18 years	18 (14.0)	23 (13.6)	
NCI risk classification			
Standard Risk	69 (53.5)	73 (43.2)	0.08
High Risk	60 (46.5)	96 (56.8)	
WHO-HEM5 Classification			
B-ALL with *BCR::ABL1* fusion	4 (3.1)	8 (4.7)	
B-ALL with *KMT2A* rearrangement	4 (3.1)	1 (0.6)	
B-ALL with *ETV6::RUNX1* fusion	6 (4.7)	3 (1.8)	0.11
B-ALL/with *IGH::IL3* fusion	1 (0.8)	1 (0.6)	
B-ALL with *TCF3::PBX1* fusion	4 (3.1)	1 (0.6)	
B-ALL, NOS**	110 (85.2)	157 (91.7)	
WBC at diagnosis			
<50 x 10^9^/L	104 (80.6)	122 (72.2)	0.09
≥50 x 10^9^/L	25 (19.4)	47 (27.8)	
CNS3 at initial diagnosis			
Yes	8 (6.2)	19 (11.2)	0.13
No	121 (93.8)	150 (88.8)	
Testicular/Ovarian involvement at diagnosis			
Yes	7 (5.4)	5 (3.0)	0.28
No	122 (94.6)	164 (97.0)	
Maternal Education			
< 9 years	77 (59.6)	116 (68.6)	0.21
9-12.9 years	38 (29.5)	42 (24.9)	
≥13 years	14 (10.9)	11 (6.5)	
Public Health Insurance Affiliation			
Yes	40 (31.0)	36 (21.3)	0.06
No	89 (69.0)	133 (78.7)	
Death during Induction			
No	129 (100)	165 (97.6)	0.08
Yes	0 (0.0)	4 (2.4)	
Early mortality			
No	115 (89.2)	127 (75.2)	<0.01
Yes	14 (10.8)	42 (24.8)	

*Chi-square or Fisher’s Exact test when appropriate.

B-ALL, B-acute lymphoblastic leukemia; MRD, Measurable Residual Disease; NCI, National Cancer Institute; NOS, Not otherwise specified.

WHO-HEM5 Classification: 5th edition of the World Health Organization Classification of Tumors of the Hematopoietic and Lymphoid Tissues. **Ploidy and/or gene rearrangements are not routinely performed in participant hospitals.

However, some notable trends were observed. A higher proportion of patients in the MRD group were classified as standard risk according to National Cancer Institute (NCI) criteria compared to those in the non-MRD group, although the difference was not statistically significant (53.5% *vs*. 43.2%, p = 0.08). Additionally, fewer patients in the MRD group were covered by public health insurance (31.0% *vs*. 21.3%, p = 0.06), but this difference also did not reach statistical significance.

Importantly, early mortality during the first year of treatment was significantly lower among patients who underwent MRD testing compared to those who did not (10.3% *vs*. 24.6%, p<0.01).

### Impact of MRD-based monitoring on first-year overall survival in pediatric B-ALL patients

**Figure 1:**
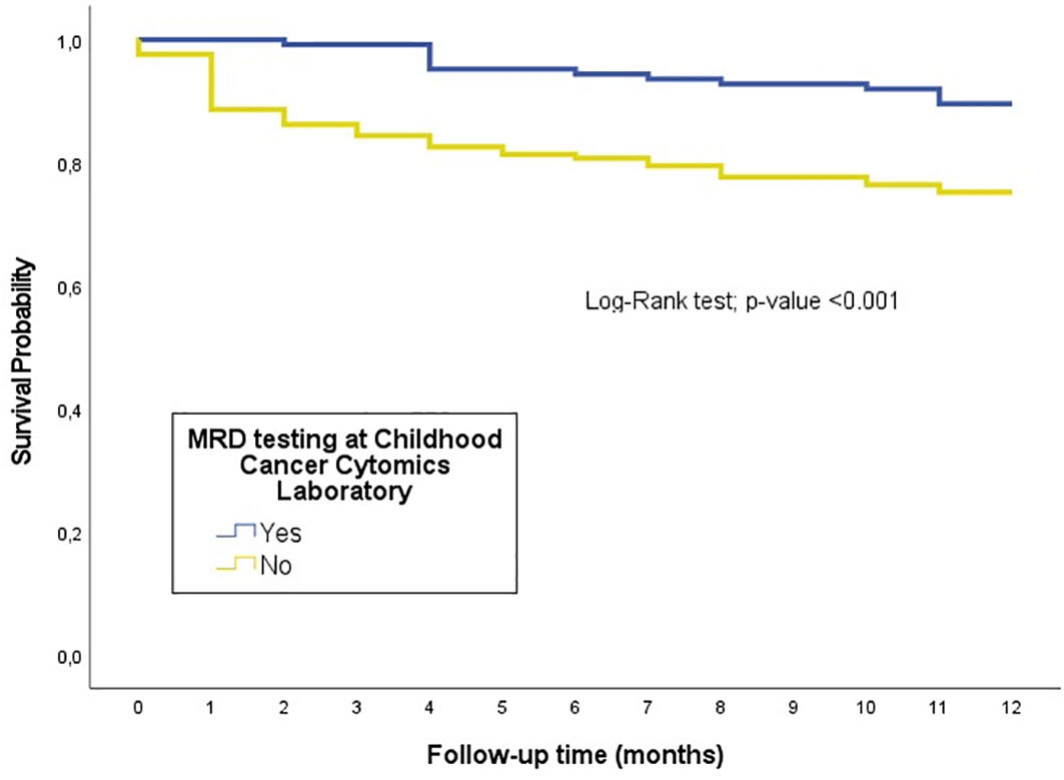
Kaplan-Meier survival curves for overall survival (OS) during the first year from diagnosis, comparing patients who underwent MRD testing at the CCCL (n=129) with those who did not (n=169). The curves illustrate the survival probabilities for each group, and differences between groups were assessed using the log-rank test.

In the first year of follow-up, patients with MRD-CCCL presented an overall survival rate of 89.6%, while those diagnosed but not adhering to follow up through MRD-CCCL had a survival rate of 75.2% (p<0.001) (**Figure 1**).

**Figure 2:**
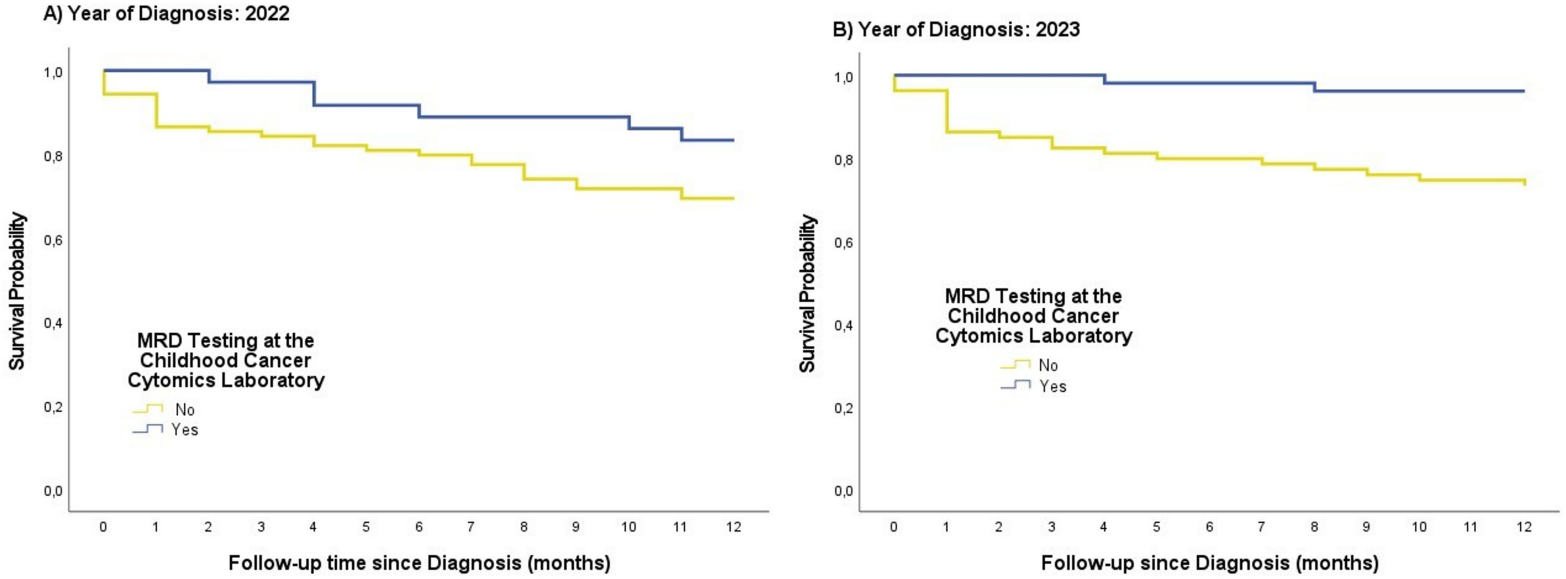
One-year overall survival (OS) from diagnosis confirmation among pediatric B-ALL patients, comparing those who underwent diagnostic evaluation and treatment response monitoring through the Childhood Cancer Cytomics Laboratory (CCCL) and those who did not, stratified by year of diagnosis. **(A)** Patients diagnosed in 2022 (n=148). **(B)** Patients diagnosed in 2023 (n=150).

Since March 2022, when the Childhood Cancer Cytomics Laboratory (CCCL) began operations with the reception of its first diagnostic sample, the first-year overall survival rates among patients diagnosed that year varied notably based on the type of monitoring received. Patients who underwent both diagnosis and treatment response monitoring through MRD testing at the CCCL (Yes) had a one-year overall survival rate of 83.3%, compared to 69.4% in those who were not (**Figure 2A**). These findings highlight the potential impact of incorporating standardized MRD-based risk re-stratification into clinical practice, suggesting improved early outcomes in the group managed through the CCCL protocol.

Moreover, as the program progressed and became more established—with improved implementation of registration protocols and standardized diagnostic and prognostic testing at the CCCL—a greater reduction in early mortality during the first year of follow-up was observed in 2023 compared to outcomes during the first year of the PRONAII Strategy (9). Notably, nearly all patients who were treated based on clinical immunophenotyping at diagnosis and subsequently re-stratified according to MRD findings survived during the first year after diagnosis confirmation (96.2%) in comparison with those not analyzed in the CCCL (73.3%) (Log-Rank Test; p-value <0.001) (**Figure 2B**).

### Early mortality and causes of death according to monitoring strategy

Early mortality during the first year of treatment occurred in 56 out of 298 patients, corresponding to an overall early mortality rate of 18.7% in the study population. Among these, a significantly lower proportion belonged to the group that underwent MRD testing at the CCCL (10.8%) compared to those monitored using standard follow-up methods (24.8%; p = 0.001).

According to the WHO-HAEM5 classification (22), early deaths occurred in patients with the BCR::ABL1 fusion (n=4) and in one patient with the B-ALL subtype carrying the TCF3::PBX1 fusion (n=1). The majority of early deaths (n=51) were observed in patients classified as B-ALL, NOS. Among patients with BCR::ABL1 fusion who were not evaluated at the CCCL, 3 out of 8 (37.5%) died early. In comparison, 1 out of 4 patients (25%) with BCR::ABL1 fusion who were evaluated at the CCCL experienced early death (p=0.99). The single patient with TCF3::PBX1 fusion belonged to the group not evaluated at the CCCL.

Importantly, among patients with B-ALL, NOS who were evaluated at the CCCL, 13 early deaths (11.8%) were recorded during the first year of treatment. In contrast, early mortality was significantly higher in the group of B-ALL, NOS patients who were not evaluated at the CCCL, with 38 early deaths (24.2%) (p=0.01).

The distribution of causes of death was comparable between the two groups, with septic shock being the leading cause, accounting for nearly half of all deaths (42.9% in the MRD-yes group *vs*. 45.2% in the MRD-No group). Other causes—including leukemic activity, hemorrhagic shock, and metabolic complications—did not differ significantly between groups (p=0.99) (**Table 2**).

**Table 2:** Distribution of early mortality and causes of death in the study population.

Study variables	MRD testing at CCCL		P-value
	Yes (n=129)	No (n=169)	
	n (%)	n (%)	
Early Mortality (1st. Year of Treatment)	14 (10.3)	42 (24.6)	0.001
Causes of death			
Septic shock	6 (42.9)	19 (45.1)	0.99
Hemorrhagic shock	2 (14.2)	6 (14.6)	
Leukemic activity	4 (28.7)	11 (26.1)	
Other causes	2 (14.2)*	6 (14.2)**	

*Neurogenic shock and acute renal failure (n=1), intracranial hemorrhage n=(1); **Intracranial hemorrhage (n=1), Acute renal failure (n=2); severe myelosuppression; metabolic acidosis (n=2) and mixed hemorrhagic and septic shock (n=1). CCCL, Childhood Cancer Cytomics Laboratory.

Early relapse within the first year was observed in 38 patients (12.8%) from the cohort (n=298). Of these, ten experienced early mortality; three of the patients who relapsed and died early were in the group that underwent MRD testing at the CCCL, while seven were in the group that did not undergo MRD testing at the CCCL. The primary site of relapse was the bone marrow, accounting for 80% of cases (n=30), while the remaining 20% (n=8) presented as isolated central nervous system (CNS) relapse.

### Multivariable analysis of early mortality

To further explore factors independently associated with early mortality, a multivariable Cox regression analysis was performed including MRD monitoring at CCCL, sex, NCI risk classification, treatment abandonment, and early relapse. The results showed that MRD monitoring at the CCCL remained a significant protective factor against early death, even after adjusting for other clinical variables (adjusted hazard ratio [aHR] 0.41; 95% CI: 0.22–0.77; *p* < 0.01). High NCI risk classification was the only other variable significantly associated with increased risk (aHR 3.02; 95% CI: 1.61–5.66; *p* < 0.01). Sex, treatment abandonment, and early relapse were not independently associated with early mortality in the adjusted model. These findings are summarized in **Table 3**.

**Table 3:** Multivariable Cox regression analysis for early mortality events and the impact of MRD at CCCL.

Variable	Early mortality events (n=56)	HR (95% CI)	aHR (95% CI)	P-value
	n			
MRD testing at CCCL				
No (ref.)	42	––––––	––––––	
Yes	14	0.37 (0.20-0.69)	0.41 (0.22-0.77)	<0.01
Patient’s sex				
Female (ref.)	23	––––––	––––––	
Male	33	1.22 (0.71-2.10)	1.19 (0.69-2.05)	0.53
NCI risk				
Standard (ref.)	13	––––––	––––––	
High	43	3.20 (1.71-5.98)	3.02 (1.61-5.66)	<0.01
Treatment abandonment				
No (ref.)	53	––––––	––––––	
Yes	3	0.73 (0.23-2.36)	0.59 (0.17-1.95)	0.41
Early relapse				
No (ref.)	46	––––––	––––––	
Yes	10	1.49 (0.75-2.97)	1.55 (0.76-3.17)	0.22

HR, Hazard ratios; aHR, adjusted HR; 95% CI, 95% confidence interval.

MRD, measurable residual disease.

CCCL, Childhood Cancer Cytomics Laboratory.

NCI, National Cancer Institute.

### Immunophenotypic subtyping of B-ALL and its association with MRD

The immunophenotyping data collected at the CCCL facilitated the qualitative subclassification of B-ALL cases based on the expression of CD10, IgM, and cytoplasmic Kappa/Lambda light chains (cyIgK/cyIgL), following the classification criteria established by the European Group for the Immunological Characterization of Leukemias (EGIL). According to this system, 5.4% of cases were identified as Pro-B (B-I), 93.0% as Common B-ALL (B-II), and 1.6% as Pre-B (B-III). In addition to this subclassification, further characterization was performed to define three additional B-ALL subtypes based on the expression of the stemness marker CD34.

Among the 129 diagnosed cases, 49.6% were classified as the ProB subtype (CD34^+^), while 34.9% exhibited heterogeneous or bimodal CD34 expressions and were assigned to the ProB-PreB subtype. The remaining 15.5% lacked CD34 expression and were categorized as PreB (**Table 4**).

**Table 4:** Distribution of B-ALL cases by CD34-based immunophenotypic subtype and induction-phase MRD status in patients analyzed at the CCCL.

Study variables	Total patients analyzed in the CCCL (n=129)			P-value
	ProB	ProB-PreB	PreB	
	n (%)	n (%)	n (%)	
B-ALL subtype	64 (49.6)	45 (34.9)	20 (15.5)	<0.001
MRD result				
Detectable (0.01-1%)	22 (34.4)	9 (20.0)	2 (10.0)	0.03*
Undetectable (<0.01)	42 (65.6)	36 (80.0)	18 (90.0)	

*Fisher exact test.

During follow-up in the induction-to-remission phase, MRD was detected in 25.5% of patients. Analysis of MRD distribution by subtype revealed that 66.6% of MRD-positive cases (22/33) belonged to the ProB group, suggesting greater resistance to initial intensive chemotherapy in this subtype. In contrast, the CD34-negative PreB subtype demonstrated a significantly lower incidence of MRD (p=0.04), indicating a more favorable early response to treatment.

No statistically significant differences in early mortality (within the first year of treatment) were observed across the subtypes. These findings highlight the clinical relevance of CD34 expression not only for subtyping and categorization at diagnosis but also as a potential prognostic marker. Incorporating CD34, along with other biological variables, into future risk stratification models could enhance the prediction of unfavorable outcomes during the first year of therapy in B-ALL.

## Discussion

This study provides evidence that the implementation of standardized immunophenotyping and MRD monitoring during induction therapy is associated with a significant reduction in early mortality among children with B-ALL treated in vulnerable regions of southern Mexico. Early mortality was lower in patients who underwent MRD testing at the CCCL compared to those who did not (10.3% *vs*. 24.6%, p < 0.01). These findings are particularly relevant in LMICs, where early treatment-related deaths continue to pose a significant barrier to survival, and where the implementation of diagnostic and prognostic tools, such as immunophenotyping and flow cytometry-based MRD monitoring, could have a substantial clinical impact and save lives (23–26).

The observed reduction in early mortality was driven by the clinical use of MRD results to guide treatment decisions. In all cases, MRD assessments at the CCCL were communicated to attending physicians within 72 hours, enabling timely and actionable clinical interventions: patients with positive MRD received post-induction therapy intensification and closer monitoring, whereas MRD-negative patients continued standard treatment protocols. Importantly, MRD testing at EOI not only stratifies patients by relapse risk but also informs decisions about therapy de-escalation or intensification. In the Vora et al. trial, MRD-positive patients benefited significantly from augmented post-remission therapy, resulting in improved event-free survival (27). Similarly, Moorman et al. reported that therapy could be safely reduced for low-risk patients identified by MRD negativity, demonstrating the utility of MRD as a bidirectional decision-making tool (28).

In addition, our findings demonstrate that patients who underwent MRD monitoring at the CCCL experienced a 59% reduction in the risk of early mortality (adjusted hazard ratio [aHR] 0.41, 95% CI: 0.22–0.77, p < 0.01), after adjusting for sex, NCI risk group, treatment abandonment, and early relapse. In line with our findings, the prognostic significance of MRD at the EOI has been firmly established across numerous pediatric ALL studies. International trials have demonstrated that MRD status at this critical juncture serves as one of the most powerful predictors of long-term outcomes—often surpassing conventional clinical and cytogenetic markers in multivariate analyses (12, 29).

Building on this context, it is important to highlight that a reduction in early mortality was particularly evident among pediatric patients classified as B-ALL, NOS—a group that includes both cases without recurrent genetic alterations after comprehensive molecular testing and those for whom such genetic evaluations were not performed due to limited resources or infrastructure in the participating hospitals. Given these challenges, centralized and standardized MRD assessment serves as a critical surrogate biomarker for risk stratification. MRD monitoring allows identification of high-risk patients who may benefit from treatment intensification, as well as low-risk patients who are responding adequately to therapy, thereby minimizing overtreatment. This strategy effectively addresses diagnostic limitations imposed by resource constraints and infrastructural challenges, enabling more precise, risk-adapted treatment decisions and improved clinical outcomes.

Additionally, this robust association supports the critical role of MRD surveillance as a prognostic tool and reinforces the importance of implementing MRD-guided clinical decision-making protocols to improve outcomes in pediatric patients with B-ALL. In large cohorts, such as the UKALL2003 and those reported by the Children’s Oncology Group, patients with undetectable MRD at EOI consistently exhibit superior event-free and overall survival compared to those with persistent disease (27, 28, 30). Studies from St. Jude Children’s Research Hospital have demonstrated the reliability and clinical value of both flow cytometry and PCR-based MRD assays, which show high concordance and prognostic relevance (31, 32). Our use of flow-based MRD monitoring allowed for the identification of high-risk patients even in the absence of full molecular profiling—an important advantage in low- and middle-income countries (LMICs), where genetic testing may be unavailable or inconclusive.

Interestingly, the most common cause of death in both groups was septic shock, highlighting the persistent need to strengthen infection control and intensive care capacity in tandem with diagnostic advancements (33, 34). The primary cause of death identified in our cohort was septic shock, accounting for 46.3% of total deaths. This was predominantly associated with the severe manifestation of infectious events during the first year of treatment. It is widely recognized that infections are the leading cause of mortality in children with B-ALL (11, 35–37). The severe impact of infections is particularly pronounced during induction therapy, primarily due to the profound and sustained neutropenia experienced by these patients. This condition renders them highly susceptible to bacterial, fungal, and viral infections, as well as those arising from their own microbiota or nosocomial pathogens acquired during hospitalization. Furthermore, the risk is significantly exacerbated by clinical manifestations like mucositis and immunodeficiency induced by chemotherapy, particularly the administration of corticosteroids and the persistence of bone marrow aplasia (36).

Therefore, it is essential to implement measures to reduce the increasing mortality and morbidity caused by infections in our population. On the one hand, it is crucial to consider the biological factors that negatively impact the response to pathogens, such as immune system reconfiguration during remission induction and the recovery of neutrophil populations (38). Additionally, a thorough analysis of the application of clinical protocols tailored to the population is necessary. These should include evaluating the benefits of antimicrobial therapies, such as empiric broad-spectrum antibiotics versus de-escalation strategies, with the aim of minimizing prolonged antibiotic exposure, drastic alterations to the microbiome, and the selection of super-resistant microorganisms (36, 39). Furthermore, it is imperative to implement measures within our healthcare systems that address these early stages of treatment. Infection control tools on quality (40) and best practices are critical to minimizing the risk of such adverse events (41).

Our perspective also emphasizes the value of detailed immunophenotyping as a complementary tool to MRD. The use of CD34 expression at diagnosis allowed for a practical subtyping of B-ALL blasts into ProB, ProB-PreB, and PreB groups, which correlated with MRD positivity. In particular, the ProB (CD34^+^) subtype was significantly more likely to present with detectable MRD after induction, suggesting a more resistant leukemic profile. In contrast, the PreB (CD34^−^) subtype was associated with lower MRD detection rates and potentially more favorable responses to initial chemotherapy.

A study by the Nordic Society of Pediatric Hematology and Oncology (NOPHO) in Denmark analyzed 200 B-ALL patients, identifying the CD34^+^/CD38^dim/+^TdT^dim/+^ immunophenotype as a predictor of poor induction response and MRD positivity post-induction. In this study, CD34 expression correlated with increased relapse risk and progressively increased from diagnosis to relapse. CD34^+^ leukemias exhibited overexpression of genes associated with stemness, migration, adhesion, and survival. This study was the first to establish that a CD34^-^negative immunophenotype is a favorable prognostic factor in B-ALL (42). These findings are consistent with the results previously reported by our research group, where the distinction between B-ALL subtypes based on CD34 expression has been closely associated with a substantial and functional reduction in the percentage of normal B-cell progenitors and precursors (43). Also, a high relative risk of MRD has also been identified in patients aged 10–18 years with the ProB CD34^+^ B-ALL subtype (44).

An important consideration, given our limited access to robust molecular assays, is the challenge of identifying key genetic subtypes for precise risk stratification. This difficulty is further compounded by the low positivity rate among patients undergoing molecular screening, with only 10.4% exhibiting detectable genetic alterations (**Table 2**). These limitations pose significant reductionism in disease characterization. In this context, standardized multiparametric flow cytometry has emerged as a valuable complementary tool, integrating immunophenotypic markers with established prognostic significance to enhance molecular analysis.

Studies performed in B-ALL at diagnosis have shown that CD34 antigen expression was independent of conventional risk factors, such as cytogenetic group, white blood cell count, and age. Certain cytogenetic subgroups, including hyperdiploidy and *B-other* cases, exhibited distinct CD34 expression profiles; however, these differences were not statistically significant (42). Additionally, Kulis et al. applied machine learning methods to identify antigen expression patterns that reveal potential genetic abnormalities in B-ALL cases. The combination of low antigen expression levels, particularly of CD10, CD34, and TdT, has been associated with the occurrence of *KMT2A* rearrangements, suggesting differential antigen expression as additional markers for disease characterization (45). The deep analysis of expression levels of various markers provided by the standardized immunophenotype, like CD10, CD66c, and CD9, and their relationship with CD34 may serve as a valuable tool for risk stratification at the time of diagnosis. However, its potential association with other molecular modifications has not yet been explored in our population.

These observations align with other made by large international leukemia consortia, including the European Group for the Immunological characterization of leukemias (EGIL) and the Current National Comprehensive Cancer Network (NCCN), which offer immunophenotypic frameworks (e.g., BI–BIII, Pro-B to Mature B-ALL) based on immunophenotype data (46–48). However, the practical application of these systems and others like International Consensus Classification (ICC) by the World Health Organization (WHO) in resource-limited settings is challenged by the limited availability of genetic and cytogenetic testing (49). In our context, immunophenotyping served not only as a diagnostic aid but also as a surrogate for biological behavior, particularly when molecular data were unavailable. The integration of immunophenotypic markers like CD34 could therefore enhance risk stratification strategies in under-resourced populations.

### Strengths and limitations of the study

This study offers valuable insights derived from a prospectively followed cohort of patients with B-ALL across multiple centers, reflecting real-world clinical practice. The prospective design allowed for systematic data collection, close monitoring of treatment response, and timely identification of adverse events, enhancing the reliability of the outcomes. Furthermore, the low rate of loss to follow-up (8%)—mostly attributable to treatment abandonment—underscores the robustness of the dataset, especially considering the challenges of long-term monitoring in resource-constrained settings.

To address the possibility of selection bias, we compared baseline high-risk clinical and biological characteristics between patients who underwent MRD testing at the CCCL and those who did not. Key prognostic factors—including elevated white blood cell count (≥50,000/mm³), age categories associated with high risk (≥10 years or <1 year), CNS involvement at diagnosis, presence of extramedullary disease such as testicular infiltration, and relevant molecular and genetic abnormalities—were similarly distributed across both groups without statistically significant differences (**Table 2**). Although the study was observational and did not employ randomization or matching, the comparable baseline risk profiles suggest that selection bias is unlikely to explain the observed reduction in early mortality in the MRD-tested group at the CCCL. A key methodological strength of our study is that patient grouping based on MRD assessment was retrospective and determined solely by sample availability, not by prospective clinical selection. This approach reduces the likelihood of selection bias, increasing the validity of comparisons between groups. While logistical and clinical challenges influenced sample submission, all patients received standard-of-care treatment within public hospitals and were included in the analysis, supporting the generalizability of our findings in resource-limited settings. Nonetheless, unmeasured variables such as differences in institutional resources, supportive care availability, or adherence to protocols may have influenced outcomes. Lastly, the current analysis focused on the first-year post-diagnosis; extended follow-up is needed to evaluate the impact of the strategy on relapse, late toxicities, and long-term survival rates.

### Future directions and implications for practice

Future work should focus on validating this immunophenotypic-MRD approach in larger, prospective cohorts across multiple institutions. Studies should also explore how integrating additional markers and molecular features into immunophenotypic profiling could refine risk stratification and predict treatment resistance earlier. Operational research evaluating the cost-effectiveness and implementation barriers of MRD-guided treatment in LMICs will be critical to scaling these strategies. Moreover, programs like CCCL can serve as models for how centralized diagnostic laboratories might reduce outcome disparities through standardized care pathways.

## Conclusion

This study demonstrates that the implementation of standardized immunophenotyping and MRD monitoring during induction therapy is associated with a significant reduction in early mortality among pediatric patients with B-ALL in southern Mexico—an area marked by pronounced socioeconomic vulnerability and limited access to specialized healthcare. The integration of these tools through a centralized platform, such as the CCCL, not only facilitated timely risk stratification and individualized treatment decisions but also improved first-year overall survival, particularly during the second year of the program’s implementation.

Although the study was observational and non-randomized, the prospective design, low loss to follow-up, and consistent methodology across centers enhance the validity of the findings. The marked decrease in early mortality—from 24.6% in patients without MRD monitoring to 10.3% in those who received it—underscores the transformative potential of applying even relatively basic diagnostic technologies in resource-limited settings. Furthermore, the association between CD34-based immunophenotypic subtypes and MRD status suggests that simple immunologic profiles may serve as practical proxies for risk classification in the absence of molecular testing.

In conclusion, the results support the feasibility and clinical relevance of MRD-guided treatment in low- and middle-income settings, and they highlight the value of tailored diagnostic infrastructure to reduce health disparities in childhood leukemia. Expanding access to standardized immunophenotyping and MRD monitoring could represent a crucial step toward improving survival outcomes in vulnerable pediatric populations across Mexico and similar regions globally.

## Data Availability

The raw data supporting the conclusions of this article will be made available by the authors, without undue reservation.
