# Supplementary material for: Impact of standardized immunophenotyping and MRD monitoring on early mortality reduction in childhood leukemia: a step towards addressing healthcare disparities in vulnerable populations from Southern Mexico
Source: Front Oncol. 2025 Jul 28;15:1614445. doi: 10.3389/fonc.2025.1614445 (PMC12336491; doi:10.3389/fonc.2025.1614445)
Supplement: Supplementary file 1 [file DataSheet1.docx]

**Supplementary material.**

Table 1. Analyzed Euroflow™ staining panels for diagnosis of B-ALL and detection of measurable/minimal residual disease (MRD).

|  | Tube number | Fluorochromes by BD Biosciences products | | | | | | | |
| --- | --- | --- | --- | --- | --- | --- | --- | --- | --- |
|  |  | BD Horizon™ V450 | BD Horizon™ V500-C | FITC | PE | PerCP-Cy™5.5 | PE-Cy™7 | APC | APC-H7 |
| ALOT* | 1 | cyCD3  clone UCHT1 #560365 | CD45  clone 2D1  #647449 | cyMPO  clone 5B8  #340580 | cyCD79a  clone HM47  #340579 | CD34  clone 8G12 #347203 | CD19  clone SJ25C1  #341093 | CD7  clone M-T701 #653311 | smCD3  clone SK7 #641397 |
| BCP-ALL extended panel* | 1 | CD20  clone L27 #642274 | CD45  clone 2D1  #647449 | CD58  clone 1C3  #55920 | CD66c  clone  B6.2/CD66  #51478 | CD34  clone 8G12 #347203 | CD19  clone SJ25C1  #341093 | CD10  clone HI10a  #340923 | CD38  clone HB7  #656646 |
|  | 2 | smIgκ  clone TB28-2 #658286 | CD45  clone 2D1  #647449 | cyIgμ  Clone  G20-127  #555782 | CD33  clone P67.6 #347787 | CD34  clone 8G12 #347203 | CD19  clone SJ25C1  #341093 | Igμ  clone G20-127 #551062 CD117  clone 104D2 #341096 | cyIgλ  clone 1-155-2  #656648 |
|  | 3 | CD9  clone M-L13 #658167 | CD45  clone 2D1  #647449 | nuTdT  clone E17-1519  #347194 | CD13  clone L138 #347837 | CD34  clone 8G12 #347203 | CD19  clone SJ25C1  #341093 | CD22  clone S-HCL-1 #340933 | CD24  clone ML5  #658331 |
|  | 4 | CD21  clone B-ly4 #658169 | CD45  clone 2D1  #647449 | CD15  clone MMA  #347423 | NG2  clone 9.2.27  #582415 | CD34  clone 8G12 #347203 | CD19  clone SJ25C1  #341093 | CD123  Clone 9F5 #658171 | CD81 clone JS-81  #656647 |
| BCP-ALL MRD* | 1 | CD20  clone L27 #655872 | CD45  clone 2D1  #647449 | CD81  Clone JS-81  #551108 | **CD66c**  Clone B6.2/CD66  #51478  **CD123**  Clone 9F5  #340545 | CD34  clone 8G12 #347203 | CD19  clone SJ25C1  #341093 | CD10  clone HI10a  #340923 | CD38  clone HB7  #656646 |
|  | 2 | CD20  clone L27 #655872 | CD45  clone 2D1  #647449 | CD81  Clone JS-81  #551108 | **CD73**  Clone AD-2  #550257  **CD304**  Clone U21-1283  #565951 | CD34  clone 8G12 #347203 | CD19  clone SJ25C1  #341093 | CD10  clone HI10a  #340923 | CD38  clone HB7  #656646 |

ALOT: Acute Leukemia Orientation Tube

BCP-ALL: B cell precursor acute lymphoblastic leukemia

MRD: Measurable/minimal residual disease

*B-ALL diagnosis and MRD quantification are both standardized methodology of the EuroFlow consortium.

**References:**

Kalina, T., et al. (2012). "EuroFlow standardization of flow cytometer instrument settings and immunophenotyping protocols." Leukemia **26**(9): 1986-2010.

Theunissen, P., et al. (2017). "Standardized flow cytometry for highly sensitive MRD measurements in B-cell acute lymphoblastic leukemia." Blood **129**(3): 347-357.

|  | **2-dimentional dot-plots with flow cytometry for ALOT tube** | | | | |
| --- | --- | --- | --- | --- | --- |
| CD34 subtype B-ALL | SSC-A/CD45 | CD19/CD34 | CD19/cyCD79a | CD7/cyMPO | cyCD3/smCD3 |
| 1. **ProB**   **CD34+** | 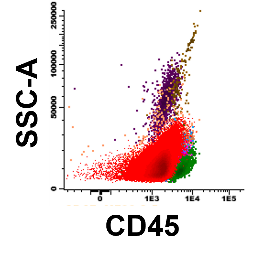 | 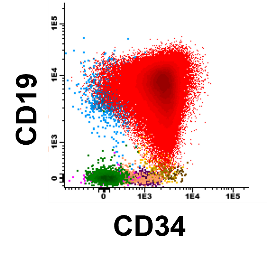 | 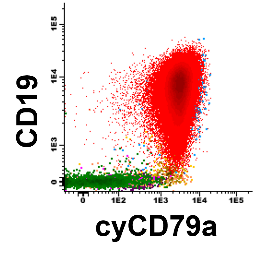 | 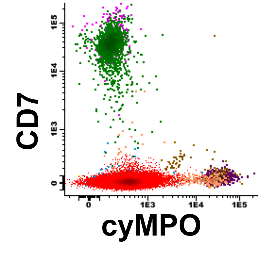 | 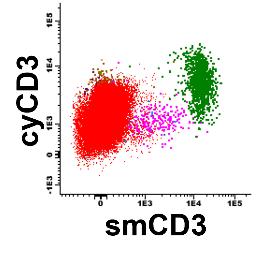 |
| 1. **ProB-PreB**   **CD34^+/-^** | 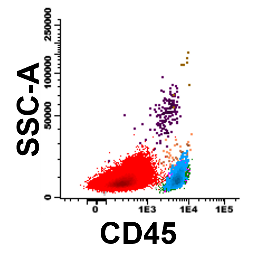 | 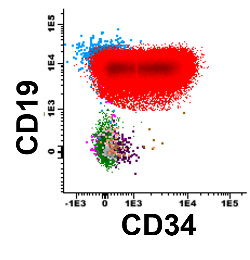 | 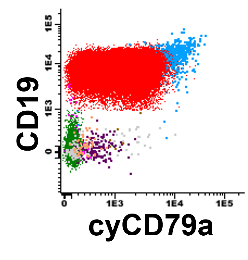 | 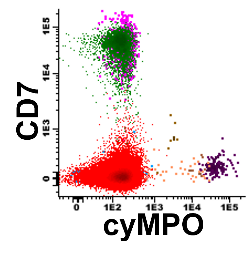 | 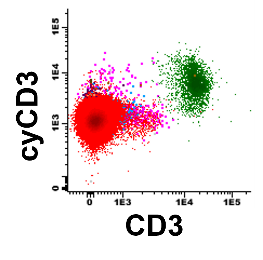 |
| 1. **PreB**   **CD34^-^** | 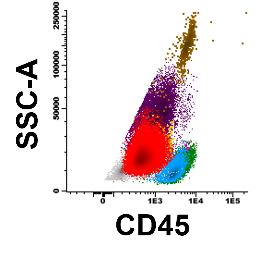 | 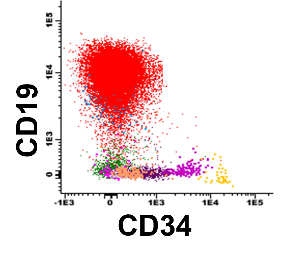 | 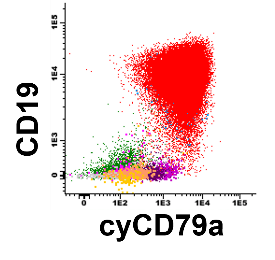 | 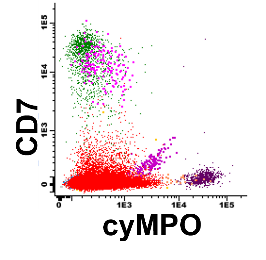 | 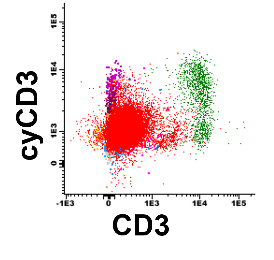 |

**Figure S1** **Gating strategy for malignant populations for recognition of B cell acute lymphoblastic leukemia in bone marrow staining on ALOT tube orientation.**

Dead cells/debris and doublets are removed using the dot plots FSC/SSC and FSC Area/FSC Height. The SSC-A/CD45 plot provides a useful overview of the gated populations and can be used as a visual check of the gated cells. The B-cell specific lineage markers CD19 and cyCD79a are used as primary gating marker and selected on the CD19/CD34 dot plot. The positive events are gated in a contour plot below the CD19 gate in the hierarchy. These events are classified according to their CD34 expression levels in the malignant population: A) ProB (high expression or positive, denoted as "+"); B) ProB-PreB (bimodal or heterogeneous expression of CD34, characterized by the coexistence of subpopulations with different expression levels "+/− or het") and C) PreB (low or absent expression, denoted as "-"). Expression of CD7, cyMPO, smCD3 and cyCD3 should be negative on blast. Subsequently, the expression pattern of normal B cell markers, along with the aberrant markers specified in Supplementary Table 1 of the BCP-ALL extended panel, is analyzed. This analysis enables the identification of abnormal maturation stage markers, leukemia-associated immunophenotype markers, and prognostic markers (data not shown).

Blast cells ●, mature T-cells ●, mature B-cells ●, neutrophils ●, monocytes ●, eosinophils ●, CD34+ progenitors●.

| Steps of MRD detection |  | 2-dimentional dot-plots with flow cytometry for MRD | | |
| --- | --- | --- | --- | --- |
| **Identification of CD19 positive populations** | **A** | SSC-A/CD45 | **B** | CD19/CD34 |
|  |  | 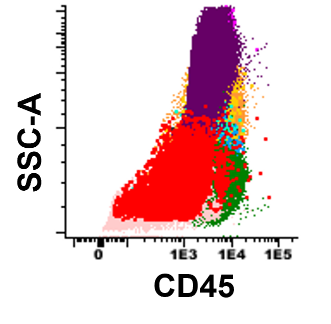 |  | 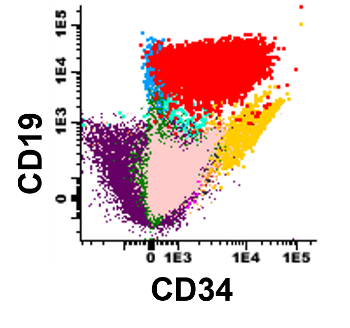 |
| **Distinction of normal B-cell reactive cells vs B-cell malignant blast** | **C** | CD20/CD10  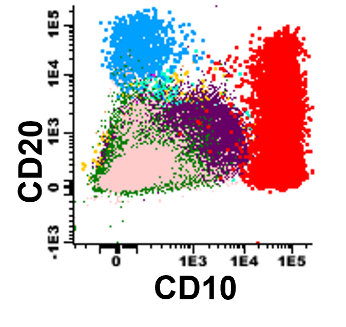 | **D** | CD38/CD81  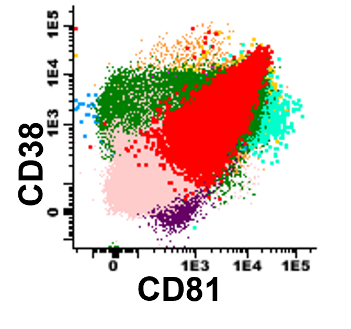 |
| **Recognition of aberrant antigens** | **E** | CD66c+CD123/CD19  **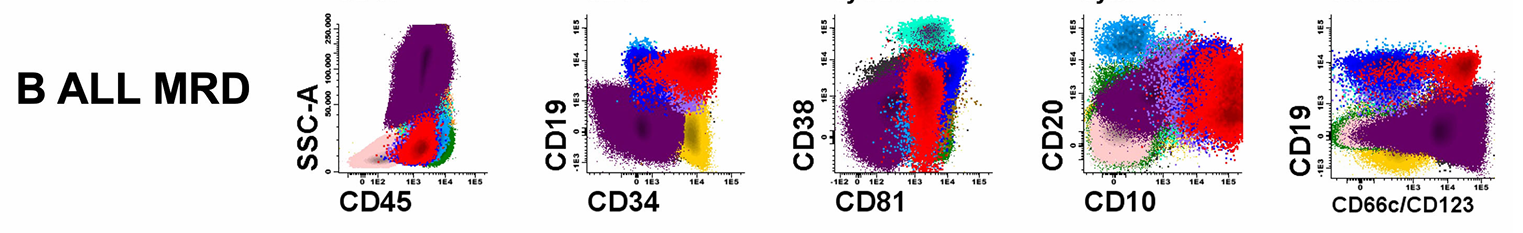** | **F** | CD73+CD304/CD19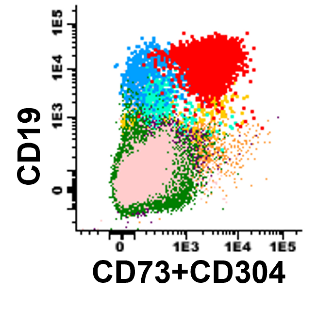 |

**Figure S2 Gating strategy for malignant populations for recognition of measurable/minimal residual disease of B-ALL according to standardized method.**

The selection of nucleated cells is recorded by eliminating dead cells, cellular debris, and doublets using the FSC/SSC and FSC Area/FSC Height scatter plots. A) Through the SSC-A/CD45 plot, clusters of recovering cell populations are identified. Residual disease has weak expression of CD45. Then, the selection of CD19 positive population is made, according to CD34 expression. B) The intensity of expression of normal B cell markers, such as CD10, CD20, CD34, CD38, CD45, and CD81, is verified to ensure compatibility with the B cell maturation stage. Residual disease exhibits partial and heterogeneous expression of CD34, positive expression of CD10 ranging from bright to moderate, heterogeneous expression of CD20 varying from negative to weakly positive, intermediate expression of CD38 and low expression of CD81. Figure C) shows the positive expression of aberrant markers CD66c/CD123 and CD73/CD304 in malignant blasts. Populations detected outside the normal maturation pattern and those exhibiting aberrant antigen expressions can be considered as positive minimal/measurable residual disease (MRD). Blast cells (red), normal B-cell precursors (green).

Blast cells ●, mature T-cells ●, mature B-cells ●, neutrophils ●, monocytes ●, eosinophils ●, CD34+ progenitors●, precursors B-cells (PreB-I) ●, precursors B-cells (PreB-II) ●, plasma cells ●, erythroid precursors ●.

.
